# Supplementary material for: Implication of the Autophagy-Related Protein Beclin1 in the Regulation of EcoHIV Replication and Inflammatory Responses
Source: Viruses. 2023 Sep 14;15(9):1923. doi: 10.3390/v15091923 (PMC10537636; doi:10.3390/v15091923)
Supplement: Supplementary file 1 [file viruses-15-01923-s001.zip › viruses-2553197-supplementary.pdf]

Implication of the Autophagy-related protein Beclin1 in the regulation of EcoHIV replication and inflammatory responses.

Myosotys Rodriguez[1], Florida Owens[1], Marissa Perry[1], Nicole Stone[1], Yemmy Soler[1], Rianna Almohtadi[1], Yuling Zhao[2,3], Elena V. Batrakova[2,3], and Nazira El-Hage[1]*

[1]Department of Immunology and Nanomedicine, Herbert Wertheim College of Medicine, Florida International University, Miami, FL 33199, USA

[2]Center for Nanotechnology in Drug Delivery, University of North Carolina at Chapel Hill, Chapel Hill, NC 27599, USA.

[3]Eshelman School of Pharmacy, University of North Carolina at Chapel Hill, Chapel Hill, NC 27599, USA

*Correspondence: Dr. Nazira El-Hage, Department of Immunology and Nanomedicine, Florida International University, Herbert Wertheim College of Medicine, Miami, FL 33199, USA; E-mail address: nelhage@fiu.edu; Phone: (305)-348-4346; Fax: (305)-348-1109.

SUPPLEMENTAL MATERIALS

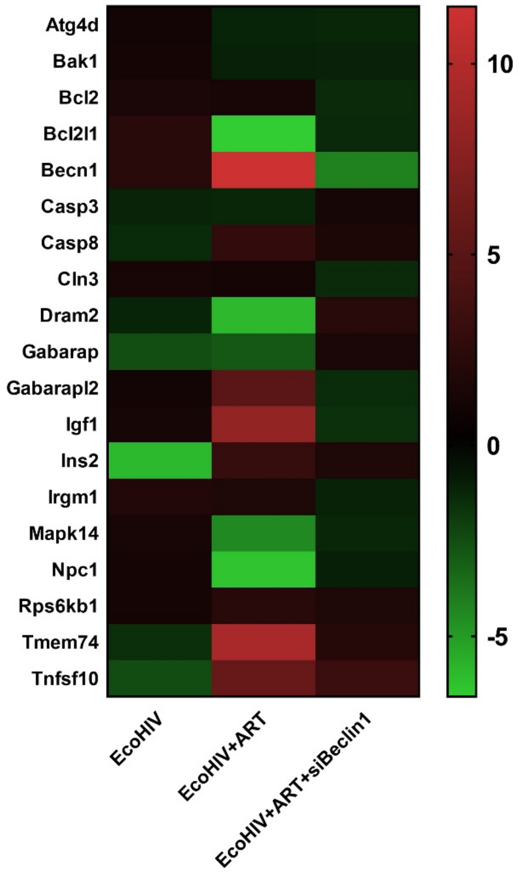

Supplemental Figure S1: Decreased mRNA expression of several autophagy-related genes in EcoHIV-infected mice after exposure to siBeclin1 in combination with ART.

Total RNA extracted from postmortem brain tissues were used to measure the expression of 84 autophagy genes using the RT2 Profiler PCR Arrays (catalog #: PAMM-084Z, Qiagen). Ct data were interpreted according to a website provided by the manufacturer. Relative expression was calculated using the ΔΔCT method with five housekeeping genes and compared with the expression in non-treated control brains. EcoHIV caused a significant increase in several genes related to the autophagy pathway including *Becn1*, *Atg4d*, *Bcl2*, *Gabarapl2*. Exposure to siBeclin1 nanoplex in combination with ART further decreased these genes.

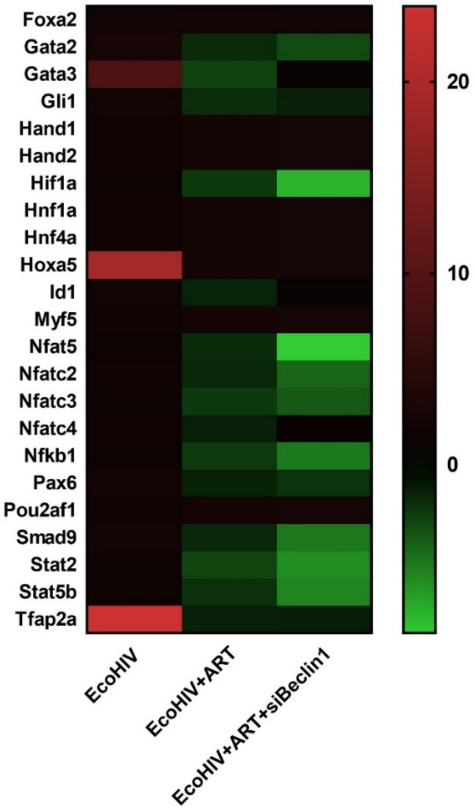

Supplemental Figure S2: Decreased mRNA expression of several transcription factors in EcoHIV-infected mice after exposure to ART alone and in combination with siBeclin1.

Total RNA extracted from postmortem brain tissues were used to measure the expression of 84 transcription factors genes using the RT2 Profiler PCR Arrays (catalog #: PAMM-075ZD, Qiagen). Ct data were interpreted according to a website provided by the manufacturer. Relative expression was calculated using the ΔΔCT method with five housekeeping genes and compared with the expression in non-treated control brains. EcoHIV caused small modifications in several transcription factors, including *NF-kB*, while most apparent was the 10-fold increase in *Gata1*, the 13-fold increase in *Hoxa5* and the 25-fold increase in *TFAP2A*, when compared to control-treated samples. Exposure to ART (consisting of lopinavir, atazanavir and abacavir) alone and in combination with the siBeclin1 nanoplex caused a 20-fold decrease in *TFAP2A*.

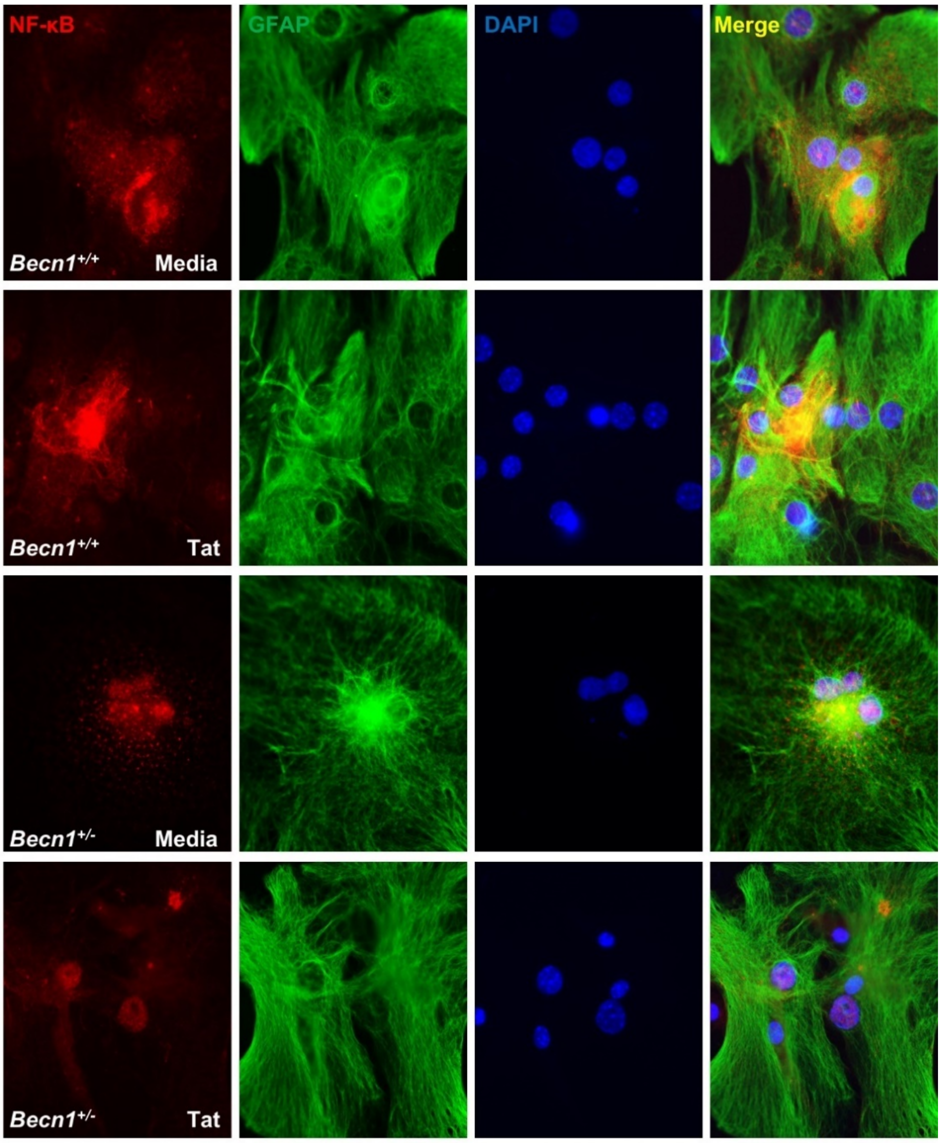

Supplemental Figure S3: NF-κB p65 expression predominantly detected in the nucleus of glia derived from *Becn1*$^{+/-}$ pups.

Primary murine glia recovered from wild-type control C57BL/6 (*Becn1*$^{+/+}$) and *Becn1*$^{+/-}$ pups at postnatal day 7, were treated with 100 nM HIV Tat for 24 hours. Cells rinsed in PBS were fixed in 4% paraformaldehyde, permeabilized, and blocked in 0.1% Triton X-100 with 10% milk/1% goat serum. Antibody against NF-κB p65 (red) (Cat#: sc-109) at 1:50 dilution and antibody against glial fibrillary acidic protein (GFAP) (green) (Cat#: MAB360) at a 1:400 dilution was used for co-immunolabelling. Cells were mounted with ProLong Gold Antifade reagent with DAPI (blue) to label cell nuclei. Images were analyzed using an inverted fluorescence microscope with a 560 Axiovision camera at 40x. Expression level of NF-κB p65 in glia derived from *Becn1*$^{+/+}$mice was

detected in the nucleus and in the cytoplasm, while expression of p65 was predominantly detected in the nucleus of glia derived from *Becn1*$^{+/-}$ mice. Exposure to Tat in C57BL/6-derived glia caused a noticeable increase in NF-κB p65 in the cytoplasm and the nucleus. On the contrary, *Becn1*$^{+/-}$-derived glia exposed to Tat did not enhance p65 expression in the cytoplasm but did enhance nuclear translocation.

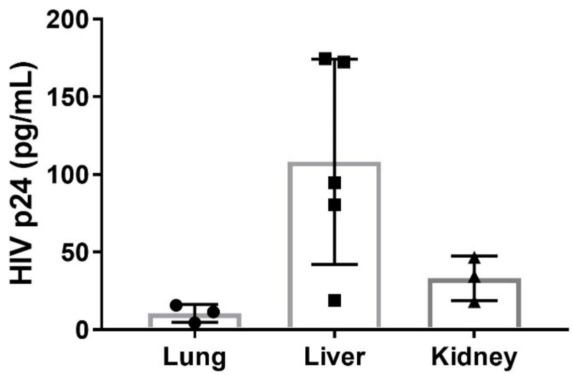

Supplemental Figure S4: p24 gag protein detected in lung, liver and kidney recovered postmortem from EcoHIV infected C57BL/6 mice.

Lung, liver and kidney recovered at necropsy were homogenized and used to measure levels of HIV p24 by ELISA, as described in Methods. The concentration of p24 detected varied among the organs, with the greatest concentration detected in the liver followed by the kidney and to a less extent, lungs. It is worth mentioning that the liver had an overall larger size volume than the rest of the organs, which may account for the greater detection of p24.

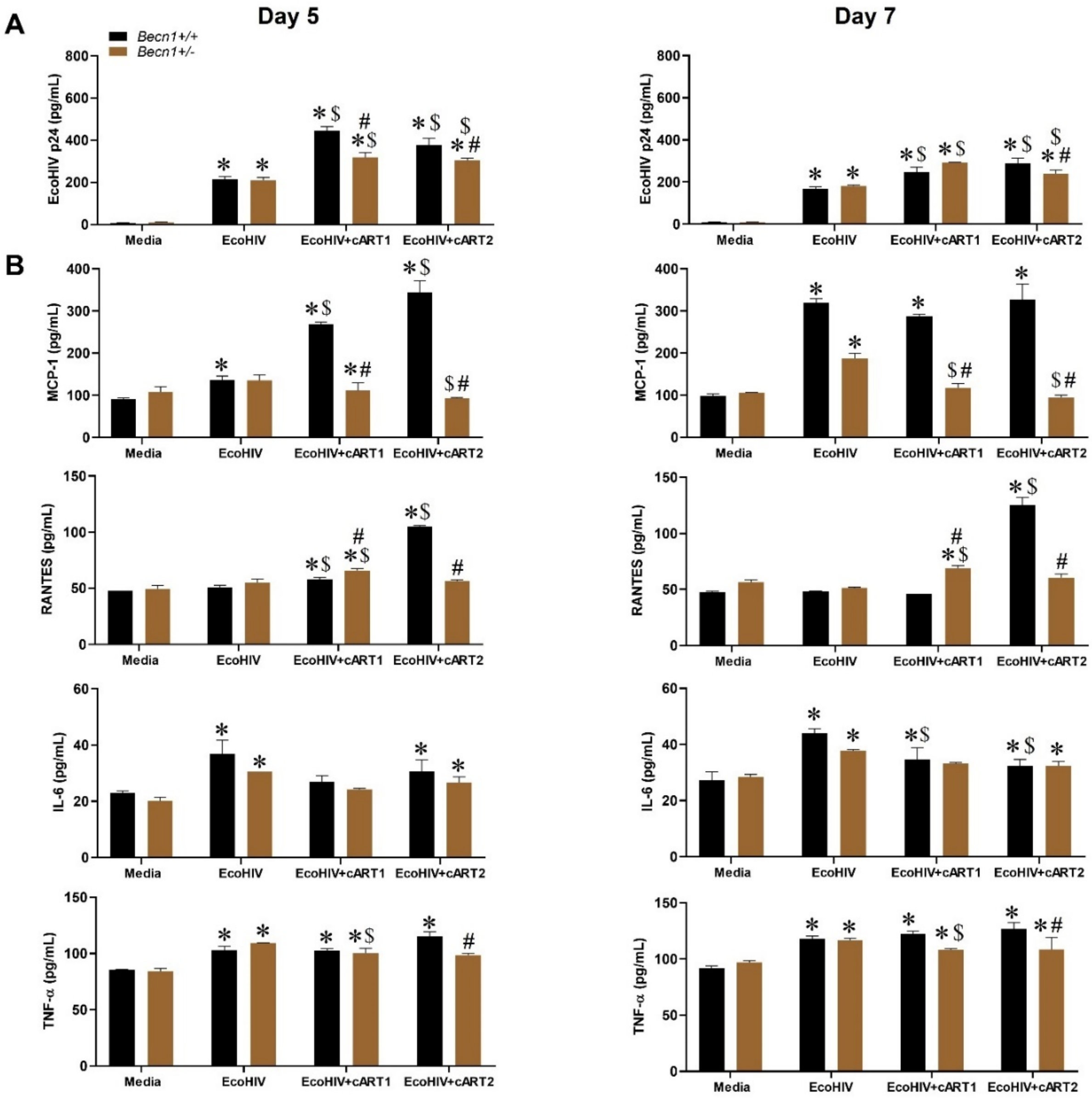

Supplemental Figure S5: Increased secretion of inflammatory molecules with combined ART predominately in glia derived from wild-type control *Becn1*$^{+/+}$ but not in *Becn1*$^{+/-}$ mice. Primary murine glia recovered from wild-type control C57BL/6 (*Becn1*$^{+/+}$) and *Becn1*$^{+/-}$ pups at postnatal day 7, were infected with 1 ng p24 Gag EcoHIV with and without exposure to 10 µM of cART1 (emtricitabine, ritonavir and atazanavir) or 10 µM of cART2 (lopinavir, abacavir, and raltegravir). After 5- (left) and 7-days (right) supernatant was removed and used to measure levels of (A) HIV p24, and (B) pro-inflammatory chemokines, MCP-1, RANTES, and pro-inflammatory cytokines IL-6, and TNF-α. After 5- days, about 200 pg/mL p24 gag protein was detected in glial

supernatant, irrespective of strain (A). Surprisingly, exposure to cART1 or cART2 in *Becn1*$^{+/+}$-derived glia caused an increase in p24 Gag (A: black bars) that correlated with an increase in MCP-1 and RANTES secretion (B: black bars). Although a similar pattern in the levels of p24 Gag protein was detected in *Becn1*$^{+/-}$ -derived supernatant after exposure to cART1 or cART2 (A: brown bars), levels of p24 Gag were lower compared to *Becn1*$^{+/+}$-derived glia. This observation correlated with 2.7-fold reduced levels in MCP-1 after exposure to cART1 (B: brown bar), 3.5-fold reduced levels in MCP-1 and a 2.0-fold decrease in RANTES after exposure to cART2 (B: brown bars) when compared to similar treated *Becn1*$^{+/+}$-derived glia (B: black bars). Similar patterns were detected after day 7 post-treatment. The secretion of IL-6 and TNF-α was increased with infection, while exposure to cART1 or cART2 had minimal effect, irrespective of glia-derived strain (B). Of note, the levels of inflammatory chemokines and cytokines were high in supernatant from cells exposed to media only. The observed increases in inflammatory molecules were mainly induced by the antiretrovirals exposure rather than the EcoHIV infection. Results are reported as the mean ± SEM of three independent experiments. Data were analyzed using one or two-way ANOVA analysis followed by Tukey's multiple comparisons test. A value of $p < 0.05$ was considered significant * vs. control counterpart, $ vs. EcoHIV counterpart, # vs. *Becn1*$^{+/+}$ counterpart.
